# A randomised sham-controlled study evaluating rTMS analgesic efficacy for postherpetic neuralgia

**DOI:** 10.3389/fnins.2023.1158737

**Published:** 2023-05-11

**Authors:** Huan Wang, Yuzhong Hu, Jiayi Deng, Yang Ye, Manli Huang, Xianwei Che, Liang Yu

**Affiliations:** ^1^Zhejiang Chinese Medicine University, Hangzhou, China; ^2^Department of Anesthesiology, Sir Run Run Shaw Hospital, College of Medicine, Zhejiang University, Hangzhou, China; ^3^Centre for Cognition and Brain Disorders, The Affiliated Hospital of Hangzhou Normal University, Hangzhou, China; ^4^Department of Mental Health, First Affiliated Hospital, College of Medicine, Zhejiang University, Hangzhou, China; ^5^The Key Laboratory of Mental Disorder Management of Zhejiang Province, Hangzhou, China; ^6^TMS Center, Deqing Hospital of Hangzhou Normal University, Deqing, China; ^7^Department of Pain, The Affiliated Hangzhou First People’s Hospital, Zhejiang University School of Medicine, Hangzhou, China

**Keywords:** postherpetic neuralgia, TMS, motor cortex, DLPFC, sleep

## Abstract

**Context:**

Postherpetic neuralgia (PHN) is a refractory neuropathic pain condition in which new treatment options are being developed. Repetitive transcranial magnetic stimulation (rTMS) may have the potential to reduce pain sensations in patients with postherpetic neuralgia.

**Objectives:**

This study investigated the efficacy on postherpetic neuralgia by stimulating two potential targets, the motor cortex (M1) and the dorsolateral prefrontal cortex (DLPFC).

**Methods:**

This is a double-blind, randomised, sham-controlled study. Potential participants were recruited from Hangzhou First People’s Hospital. Patients were randomly assigned to either the M1, DLPFC or Sham group. Patients received ten daily sessions of 10-Hz rTMS in 2 consecutive weeks. The primary outcome measure was visual analogue scale (VAS) assessed at baseline, first week of treatment (week 1), post-treatment (week 2), 1-week (week 4), 1-month (week 6) and 3-month (week 14) follow-up.

**Results:**

Of sixty patients enrolled, 51 received treatment and completed all outcome assessments. M1 stimulation resulted in a larger analgesia during and after treatment compared to the Sham (week 2 – week 14, *p* < 0.005), as well as to the DLPFC stimulation (week 1 – week 14, *p* < 0.05). In addition to pain, sleep disturbance was significantly improved and relieved by targeting either the M1 or the DLPFC (M1: week 4 – week 14, *p* < 0.01; DLPFC: week 4 – week 14, *p* < 0.01). Moreover, pain sensations following M1 stimulation uniquely predicted improvement in sleep quality.

**Conclusion:**

M1 rTMS is superior to DLPFC stimulation in treating PHN with excellent pain response and long-term analgesia. Meanwhile, M1 and DLPFC stimulation were equally effective in improving sleep quality in PHN.

**Clinical trial registration:**

https://www.chictr.org.cn/, identifier ChiCTR2100051963.

## Introduction

Postherpetic neuralgia (PHN) is a typical neuropathic pain condition affecting the lesioned skin regions following the healing of skin rashes (Pickering et al., 2014). Postherpetic neuralgia is very difficult to manage, even with the use of a variety of medications (Sampathkumar et al., 2009; Sawynok and Zinger, 2016). Of note, the response rate of medication therapy is not satisfactory, especially in older adults (Bonezzi and Demartini, 1999). Other treatment options are therefore needed, such as a vaccine for prevention (Oxman et al., 2005) and noninvasive brain stimulation technologies for pain relief (Ma et al., 2015; Attal et al., 2016; Pei et al., 2019).

Transcranial magnetic stimulation (TMS) is a safe and noninvasive form of brain stimulation. Repetitive TMS (rTMS) is capable of inducing neuroplastic changes which has been proposed for the treatment of neuropathic pain (Lefaucheur et al., 2014, 2020; Wang et al., 2023). Only two studies have specifically investigated the effects on postherpetic neuralgia, with the results indicating a clear analgesic effect of high-frequency rTMS over the motor cortex (M1) (Ma et al., 2015; Pei et al., 2019). However, these two studies were conducted by the same group and reported a short-term response rate of ~40% with pain reduction ≥25% but not the widely accepted 30% rule (Lefaucheur and Nguyen, 2019). Overall, the analgesic efficacy of motor cortex rTMS needs to be re-evaluated and improved in postherpetic neuralgia.

In addition to the M1, the dorsolateral prefrontal cortex (DLPFC) has been increasingly used as an alternative target in the management of neuropathic pain (Borckardt et al., 2009; Nardone et al., 2017; Leung et al., 2018; Che et al., 2021). The analgesic effect of DLPFC stimulation is suggested to be mediated by top-down pain modulation, with decreased activity being observed along the thalamus, midbrain and medulla (Martin et al., 2013; Taylor et al., 2013). Moreover, a line of evidence indicated that DLPFC is also able to modulate neural substrates associated with the emotional aspects of pain, such as the insular cortex and the anterior cingulate cortex (ACC; Lorenz et al., 2003; Tracey and Mantyh, 2007; Ye et al., 2022). This is directly relevant to postherpetic neuralgia in which refractory pain may lead to disappointment, anxiety and other emotional distress (Mauskopf et al., 1994; Schmader, 2002). However, a recent large trial provided compelling evidence that DLPFC was not superior to sham stimulation in pain management (Attal et al., 2021). It is worth noting that participants were mainly with traumatic/surgical nerve lesion or sensory polyneuropathy in this compelling trial, whereby a few patients with postherpetic neuralgia were enrolled.

Overall, the clinical efficacy of motor cortex rTMS on postherpetic neuralgia needs to be re-evaluated and independently investigated given the refractory nature of pain and its adverse influence on quality of life. We have also presented the first investigation of DLPFC stimulation in postherpetic neuralgia. Clinical effects were systematically assessed, including not only pain sensations but anxiety, depression and sleep quality. It is hypothesised that M1-rTMS would be particularly effective in reducing pain sensations, and the DLPFC stimulation would have a unique effect on emotional distress.

## Methods

### Participants

A power analysis based on VAS pain score was initially conducted using G*Power (Faul et al., 2009) (F tests, ANOVA: repeated measures, within-between interaction, Alpha = 0.05, Beta = 0.95, Cohen’s *d* = 0.59). Results indicated that 51 participants would ensure 95% statistical power. A total of sixty patients were enrolled considering potential dropout rates (~10%) (Attal et al., 2021).

Potential participants were recruited from the Affiliated Hangzhou First People’s Hospital. The inclusion criteria were: (1) IASP diagnosis of postherpetic neuralgia (Scholz et al., 2019); (2) at least 3 months after the onset of pain; (3) at least moderate pain intensity (≥3 assessed by visual analogue scale, VAS); (4) 18 years or older; (5) no adjustment in medication from 2 weeks before the allocation to the end of the trial; (6) capable of receiving TMS treatment and fulfilling clinical assessments.

The exclusion criteria were: (1) contradictions to TMS treatment (Rossi et al., 2011), such as metal implants or seizure; (2) severe mental disorders (HAMD ≥35 or HAMA ≥29); (3) aphasia or cognitive disorders (MMSE ≤24); (4) severe disorders caused by other conditions, e.g. tumour; (5) severe heart or lung misfunctioning or extremely weak. The withdraw criteria were changes in medication after allocation or that patients decided to withdraw from the study.

### Study overview

We conducted a double-blind, randomised, sham-controlled trial registered in the Chinese Clinical Trials registry (ChiCTR-IOR-14005304). Patients were randomly assigned to either M1, DLPFC or Sham group according to a centrally stratified computer-generated randomisation protocol. Patients received ten daily sessions in 2 consecutive weeks delivered by a trained staff (HW). Clinical assessments were performed at baseline, after first week of treatment (week 1), post treatment (week 2), 2 weeks (week 4), 1 month (week 6) and 3 months (week 14) follow-up. All assessments were performed by a single trained and blinded staff member (YH). All participants voluntarily participated in this study and signed an informed consent before the treatment. Ethical approval was obtained from the Ethics Committee of Hangzhou First People’s Hospital (IIT-20220301-0039). This study was conducted in accordance with the Code of Ethics of the World Medical Association (Declaration of Helsinki).

### TMS treatment

Each session started with the assessment of resting motor threshold (RMT), using a figure-eight coil connected to an RT-50 stimulation system (Sichuan Junjian Wanfeng Medical Equipment Co) delivering single pulses to the hand region of the M1 at 0.2 Hz. RMT was determined by the minimum intensity to evoke motor-evoked potentials (MEPs) >0.05 mV in 5/10 trials and re-examined in each session. Each rTMS session delivered 3,000 pulses at 10 Hz with 5-s trains and 25-s intervals at 100% RMT (Attal et al., 2016; Ayache et al., 2016).

rTMS was delivered either to the contralateral M1, the left DLPFC or a Sham condition. The M1 and DLPFC was located using the hotspot and Beam F3 methodology, respectively (Beam et al., 2009). The M1 target was contralateral to the painful side or corresponded to the left hemisphere in case of bilateral pain based on previous rTMS studies in analgesia (Lefaucheur et al., 2020; Attal et al., 2021; Wang et al., 2023). Meanwhile, the left DLPFC was stimulated according to most prior studies of pain (de Oliveira et al., 2014; Nardone et al., 2017; Cheng et al., 2023). The Sham stimulation was performed using a sham coil which does not produce a magnetic field but has the same appearance and auditory sensations as a real coil. Coil position was measured relative to the nasion and inion to facilitate consistent re-positioning of the coil between sessions (Chung et al., 2019; Ye et al., 2022).

### Clinical assessment

The outcome measures were reported according to the IMMPACT recommendations for chronic pain clinical trials (Dworkin et al., 2008). The primary outcome measure was pain intensity measured by visual analogue scale (VAS). The secondary outcome measures included the short-form McGill Pain Questionnaire (SF-MPQ) (Melzack, 1987), the Pittsburgh Sleep Quality Index (PSQI) (Buysse et al., 1989), Hamilton Depression Rating Scale (HAMD-24) (Hamilton, 1967), Hamilton Anxiety Scale (HAMA-17) (Hamilton, 1959) and the Mini–Mental State Examination (MMSE) (Folstein et al., 1975). Patients’ evaluation of the treatment was measured by the Patients’ Global Impression of Change (PGIC) (Guy, 1976). We have also evaluated pain response rate (pain reduction >2 or >30% in VAS) (Dworkin et al., 2008; Lefaucheur and Nguyen, 2019) and insomnia remission (<5) (Buysse et al., 1989; Aloba et al., 2007) following treatment.

Safety assessments were performed at each treatment and follow-up session. Headache and scalp discomfort were considered as mild side effects which were most common in rTMS treatment (Attal et al., 2021). Potential serious adverse effects were evaluated by monitoring patients’ vitality, physical and mental health.

### Data analysis

Intent to-treat (ITT) analysis was set to include all randomised patients. Multiple imputation algorithm was initially performed, which is a highly recommended methodology in dealing with missing data (McCoy, 2017).

### Statistical analysis

Data were analysed in SPSS (v.25.0 Chicago, Illinois, United States). Demographic variables were initially examined with one-way ANOVA or *χ*^2^ tests. A series of tests were performed to check the assumptions of using a mixed design ANOVA. Specifically, Shapiro–Wilk test was performed to check the normality of the outcome measures in different combinations of our two factors. Levene’s test for homogeneity of variances and Mauchly’s Test of Sphericity were also performed. Results validated the use of mixed ANOVA (*p*_s_ > 0.05).

Two-way mixed-design ANOVAs were then performed to examine the effects of treatment group (M1, DLPFC and Sham), time and their interaction on the primary and secondary outcome measures. Post-hoc pairwise comparisons were Bonferroni corrected at *p* ≤ 0.05. *χ*^2^ tests were performed to compare the effects on patients’ evaluation of the treatment (PGIC), and bivariate correlation analyses were used to examine the associations between outcome measures. We also performed *χ*^2^ tests to examine the relationship between pain response and PGIC.

According to the large neuropathic pain trial (Attal et al., 2021), we also calculated the Number Needed to Treat (NNT) based on no less than 50% reduction in VAS. NNT was calculated using the formula 1/(% improved with active minus % improved with sham).

## Results

### Clinical characteristics

**Figure 1:**
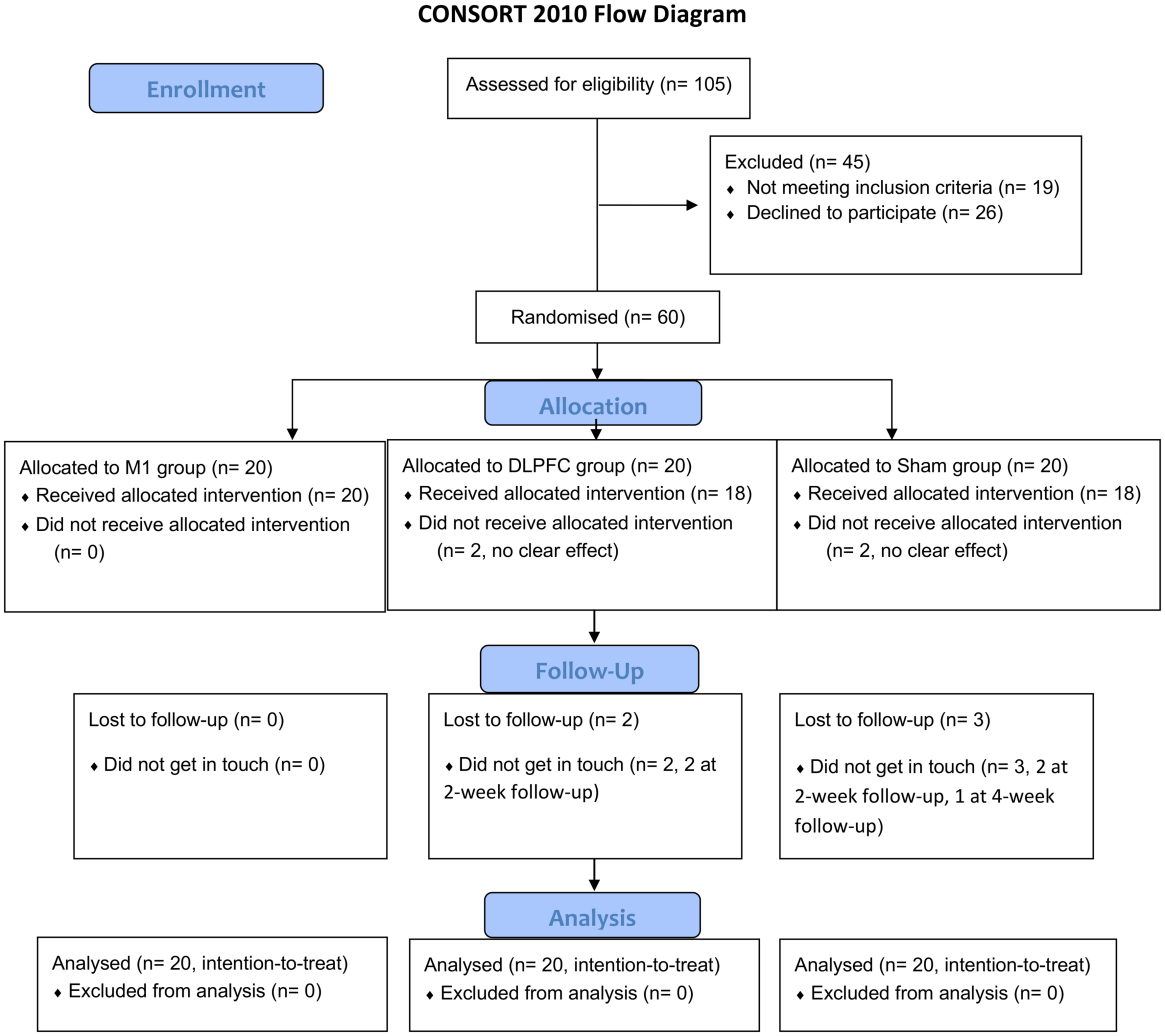
CONSORT flow diagram of this study.

A total of 105 patients were screened, of which 55 were excluded due to noting meeting the inclusion criteria (*n* = 19) or not willing to participate in (*n* = 26; Figure 1). Sixty participants were enrolled in and equally randomised to three groups. Four patients withdrew from the DLPFC group due to no clear effect (*n* = 2, within week 1) or loss of follow-up (*n* = 2, at 2-week follow-up). In the Sham group, five patients withdrew due to no clear effect (*n* = 2, within week 1) or loss of follow-up (*n* = 3, 2 at 2-week follow-up, and 1 at 4-week follow-up). All twenty patients in the M1 group were evaluated throughout. Data of twenty patients in each group were analysed with ITT methodology.

Demographic information is presented in Table 1. The pain sensations were severe (~7 in VAS), mainly located in the trunk and face/head regions. Nearly all the participants have used Pregabalin and/or Gabapentin for analgesia. Three groups showed no difference in gender, age, lesion hemisphere, predominant pain area, medication, VAS, sleep quality, HAMA, HAMD or MMSE. It is noted that the patients allocated to the M1 group had longer disease duration than the DLPFC group (*P_corrected_* = 0.044).

**Table 1:** Demographic information of participants.

Measure	M1 (*n* = 20)	DLPFC (*n* = 20)	Sham (*n* = 20)	*F*/*χ*^2^	*p*
Age, y				1.032^a^	0.363
Mean ± SD	68.50 ± 8.19	70.80 ± 9.05	67.05 ± 7.67		
Sex				5.25^b^	0.097
Male	14	12	7		
Female	6	8	13		
Lesion hemisphere				5.31^b^	0.084
Right	11	7	4		
Left	9	13	16		
Course of disease, m				3.79^a^	0.035
Mean ± SD	18.50 ± 23.57	5.65 ± 3.22	10.55 ± 14.67		
VAS				0.719^a^	0.492
Mean ± SD	7.20 ± 0.83	7.10 ± 1.12	6.85 ± 0.88		
Sleep quality				0.164^a^	0.849
Mean ± SD	10.65 ± 2.50	10.25 ± 2.22	10.50 ± 1.93		
HAMA				1.025^a^	0.365
Mean ± SD	4.25 ± 1.16	3.95 ± 1.10	3.75 ± 1.07		
HAMD				0.437^a^	0.648
Mean ± SD	4.30 ± 1.38	3.90 ± 1.48	4.15 ± 1.23		
MMSE				0.051^a^	0.950
Mean ± SD	27.65 ± 1.23	27.55 ± 1.10	27.55 ± 1.10		
Predominant pain area, *n* (%)					
Upper limbs	2 (10)	0	4 (20)		
Lower limbs	1 (5)	1 (5)	1 (5)		
Face/head	4 (20)	5 (25)	2 (10)		
Trunk	13 (65)	14 (70)	13 (65)		
Medication use, *n* (%)					
Pregabalin	16 (80)	15 (75)	15 (75)		
Gabapentin	3 (15)	5 (25)	3 (15)		
Others	1 (5)	0	2 (10)		

^a^One-way ANOVA; ^b^*χ*^2^ test; y indicates year; m indicates month.

### Treatment efficacy

**Figure 2:**
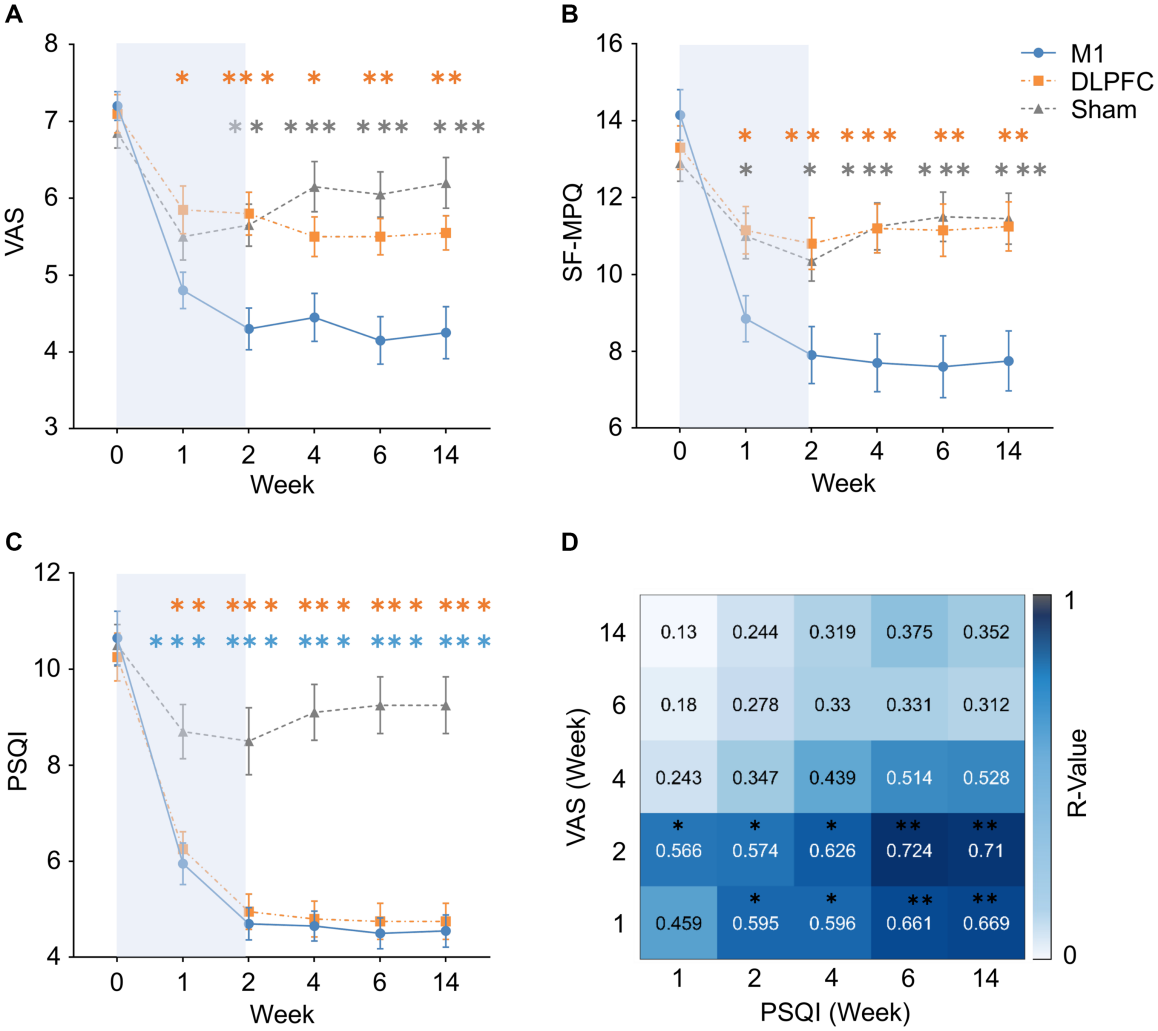
Treatment effects on pain, sleep and their association. **(A,B)** Motor cortex rTMS resulted in a larger pain reduction than the DLPFC and Sham stimulation after 1-week treatment to 3-month follow-up. DLPFC was not superior to Sham stimulation in pain sensations. Orange asterisks indicate M1-DLPFC comparisons, and grey asterisks indicate M1-Sham comparisons. **(C)** Motor cortex and DLPFC stimulation equally improved sleep quality compared to Sham stimulation after 1-week treatment to 3-month follow-up. Orange asterisks indicate DLPFC-Sham comparisons, and blue asterisks indicate M1-Sham comparisons. **(D)** Pain sensations during and after M1 treatment was able to predict sleep quality in all the follow-up periods, but not vice versa. This effect was not observed in the DLPFC condition. *P_corrected_ < 0.05, **P_corrected_ < 0.01, ***P_corrected_ < 0.001. Error bars indicate standard error of means (SEM), and the blue backgrounds indicate treatment period. VAS, Visual Analogue Scale; SF-MPQ, Short-Form McGill Pain Questionnaire; PSQI, Pittsburgh Sleep Quality Index.

In terms of the primary outcome of VAS, mixed-design ANOVA revealed a significant interaction effect (*F*_5.13, 146.19_ = 8.93, *p* = 0.001, $\eta_{p}^{2}=0.24$ ). *Post-hoc* comparisons indicated that M1 stimulation resulted a larger analgesia during and/or after treatment compared to the Sham (week 2: Mean_M1_ = 4.30, Mean_Sham_ = 5.66; *P_corrected_* = 0.002; Week 4: Mean_M1_ = 4.45, Mean_Sham_ = 6.10; *P_corrected_* = 0.001; week 6: Mean_M1_ = 4.15, Mean_Sham_ = 6.04; *P_corrected_* = 0.001; week 14: Mean_M1_ = 4.25, Mean_Sham_ = 6.18; *P_corrected_* = 0.001), as well as to the DLPFC condition (week 1: Mean_M1_ = 4.80, Mean_DLPFC_ = 5.86; *P_corrected_* = 0.035; week 2: Mean_M1_ = 4.30, Mean_DLPFC_ = 5.82; *P_corrected_* = 0.001; week 4: Mean_M1_ = 4.45, Mean_DLPFC_ = 5.50; *P_corrected_* = 0.049; week 6: Mean_M1_ = 4.15, Mean_DLPFC_ = 5.49; *P_corrected_* = 0.005; week 14: Mean_M1_ = 4.25, Mean_DLPFC_ = 5.54; *P_corrected_* = 0.011). Both the DLPFC and Sham group resulted in smaller VAS compared to the baseline (all *P_corrected_* < 0.05), but no group difference was observed between these two groups at any time (all *P_corrected_* > 0.05; Figure 2A).

In SF-MPQ, mixed-design ANOVA revealed a significant interaction effect (*F*_4.23, 120.59_ = 9.81, *p* = 0.001, $\eta_{p}^{2}=0.26$ ). *Post-hoc* comparisons indicated the same results as VAS, in which M1 stimulation resulted in less pain during and after treatment compared to the Sham (week 1: Mean_M1_ = 8.85, Mean_Sham_ = 11.00; *P_corrected_* = 0.043; week 2: Mean_M1_ = 7.90, Mean_Sham_ = 10.38; *P_corrected_* = 0.026; week 4: Mean_M1_ = 7.70, Mean_Sham_ = 11.22; *P_corrected_* = 0.001; week 6: Mean_M1_ = 7.60, Mean_Sham_ = 11.52; *P_corrected_* = 0.001; week 14: Mean_M1_ = 7.75, Mean_Sham_ = 11.44; *P_corrected_* = 0.001), as well as to the DLPFC condition (week 1: Mean_M1_ = 8.85, Mean_DLPFC_ = 11.15; *P_corrected_* = 0.028; week 2: Mean_M1_ = 7.90, Mean_DLPFC_ = 10.83; *P_corrected_* = 0.006; week 4: Mean_M1_ = 7.70, Mean_DLPFC_ = 11.20; *P_corrected_* = 0.001; week 6: Mean_M1_ = 7.60, Mean_DLPFC_ = 11.13; *P_corrected_* = 0.003; week 14: Mean_M1_ = 7.75, Mean_DLPFC_ = 11.21; *P_corrected_* = 0.003). Both the DLPFC and Sham group resulted in significant pain reduction compared to the baseline (all *P_corrected_* < 0.05), but no group difference was observed between these two groups at any time (all *P_corrected_* > 0.05; Figure 2B).

The Number Needed to Treat (NNT) analyses revealed 2.5 and 2.5 for the M1 group at post-treatment and 3-month follow-up, respectively. Meanwhile, no patients reported a pain reduction over 50% in the DLPFC group and thus NNT was not calculated.

In terms of sleep quality (i.e. PSQI), mixed-design ANOVA revealed a significant interaction effect (*F*_4.41, 125.72_ = 13.46, *p* = 0.001, $\eta_{p}^{2}=0.32$ ). *Post-hoc* comparisons indicated that, compared to the Sham stimulation, both the M1 (week 1: Mean_M1_ = 5.92, Mean_Sham_ = 8.66; *P_corrected_* = 0.001; week 2: Mean_M1_ = 4.70, Mean_Sham_ = 8.51; *P_corrected_* = 0.001; Week 4: Mean_M1_ = 4.65, Mean_Sham_ = 9.14; *P_corrected_* = 0.001; week 6: Mean_M1_ = 4.50, Mean_Sham_ = 9.20; *P_corrected_* = 0.001; week 14: Mean_M1_ = 4.55, Mean_Sham_ = 9.20; *P_corrected_* = 0.001) and DLPFC stimulation (week 1: Mean_M1_ = 6.25, Mean_DLPFC_ = 8.66; *P_corrected_* = 0.002; week 2: Mean_M1_ = 4.96, Mean_DLPFC_ = 8.51; *P_corrected_* = 0.001; week 4: Mean_M1_ = 4.83, Mean_DLPFC_ = 9.14; *P_corrected_* = 0.001; week 6: Mean_M1_ = 4.76, Mean_DLPFC_ = 9.20; *P_corrected_* = 0.001; week 14: Mean_M1_ = 4.76, Mean_DLPFC_ = 9.20; *P_corrected_* = 0.001) significantly increased sleep quality during and after the treatment (Figure 2C).

There was no effect of Group, Time or Group × Time interaction on HAMD (all *p* > 0.05), HAMA (all *p* > 0.05) or MMSE (all *p* > 0.05; Supplementary material).

In the analysis of pain response rate (pain reduction >2 or >30% in VAS), M1 stimulation resulted in larger response rates than the Sham group from post-treatment to all follow-up periods (week 2: *P_corrected_* = 0.003; week 4: *P_corrected_* = 0.003; week 6: *P_corrected_* = 0.001; week 14: *P_corrected_* = 0.001). M1 group also reported a higher response rate during the treatment (week 1, *P_corrected_* = 0.042; week 2, *P_corrected_* = 0.003) compared to the DLPFC stimulation (Table 2).

**Table 2:** Pain response and insomnia remission rates.

	Week 1	Week 2	Week 4	Week 6	Week 14
Pain response					
M1	50%a*	65%a**b**	55%b**	65%b***	65%b***
DLPFC	10%	10%	30%	30%	30%
Sham	15%	10%	5%	0%	0%
Insomnia remission					
M1	20%	45%	50%b**	60%b***	60%b***
DLPFC	10%	45%	50%c**	60%c***	60%c***
Sham	5%	10%	5%	5%	5%

a: Significant difference between M1 and DLPFC; b: Significant difference between M1 and Sham; c: Significant difference between DLPFC and Sham. *P_corrected_ < 0.05, **P_corrected_ < 0.01, ***P_corrected_ < 0.001.

In terms of insomnia remission (<5), both the M1 and DLPFC stimulation resulted in higher remission rates than the Sham group from 2-week to 3-months follow-up (M1: week 4 – week 14, *P_corrected_* = 0.009, 0.001, 0.001; DLPFC: week 4 – week 14, *P_corrected_* = 0.009, 0.001, 0.001). No group difference was observed between the M1 and DLPFC group (all *P_corrected_* > 0.05; Table 2).

**Figure 3:**
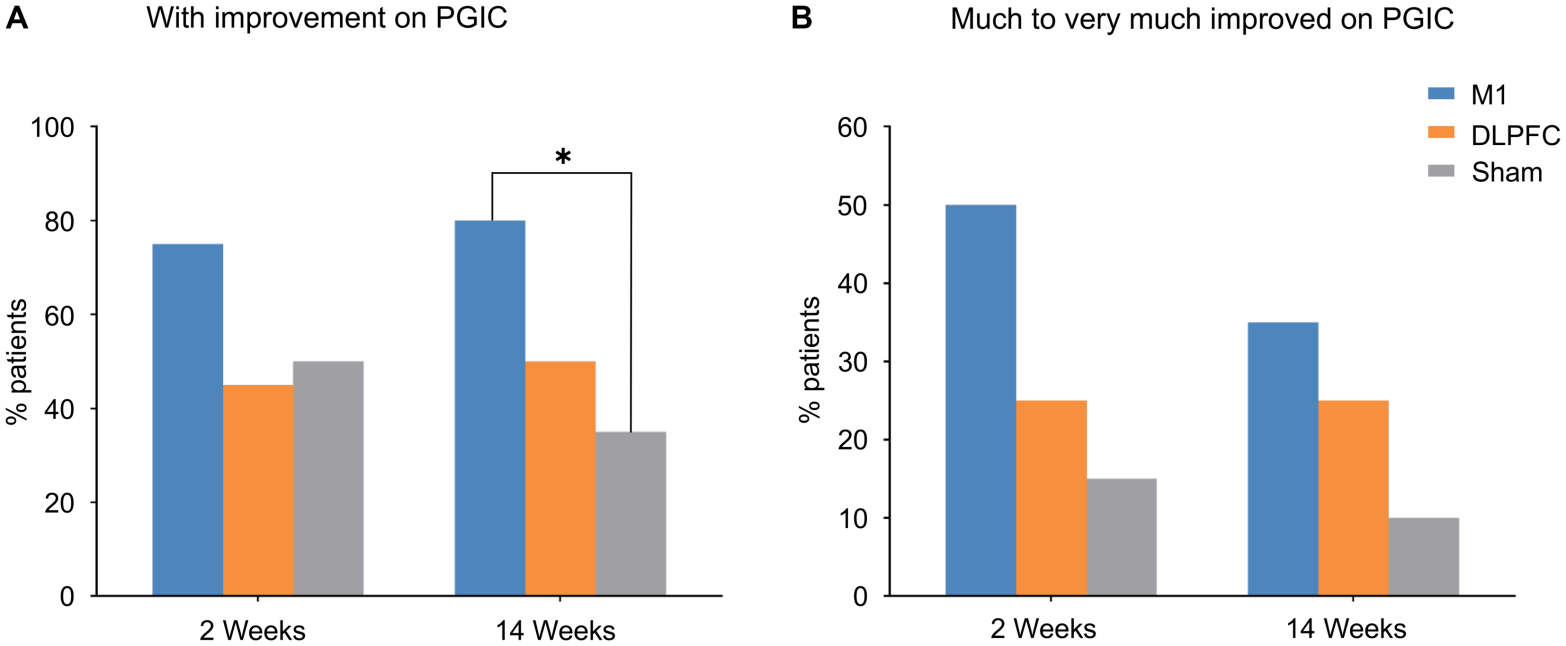
Patients’ Global Impression of Change. **(A)** More patients reported improvement in the M1 group compared to sham stimulation at 3-month follow-up. **(B)** Data on much to very much improvement revealed group difference although it did not reach statistical significance. *P_corrected_ < 0.05. PGIC, Patients’ Global Impression of Change.

In the effects on patients’ evaluation of the treatment (PGIC), *χ*^2^ tests (yes or no improvement) indicated that more patients reported improvement in the M1 group than the Sham group at 3-month follow-up (*χ*^2^ = 8.29, *P_corrected_* = 0.030; Figure 3B). This effect was not significant at post-treatment (week 2; *P_corrected_* > 0.05; Figure 3A). No other significant difference was observed between groups (all *P_corrected_* > 0.05). When data were categorised based on “much to very much improved” (Attal et al., 2021), no group difference was observed (all *P_corrected_* > 0.05; Figures 3A,B). It is noted that pain response (yes/or) was not associated with PGIC (yes/or) in either group or timepoint (all *P_corrected_* > 0.05).

### Correlation analyses

Bivariate correlation analyses corrected for multiple comparisons indicated that VAS scores during (week 1) and immediately after the treatment (week 2) were positively associated with sleep quality (i.e. PSQI) in all the follow-up periods in the M1 group (VAS_Week1_-PSQI_week4_: *P_corrected_* = 0.030; VAS_Week1_-PSQI_week6_: *P_corrected_* = 0.010; VAS_Week1_-PSQI_week14_: *P_corrected_* = 0.005; VAS_Week2_-PSQI_week4:_ *P_corrected_* = 0.012; VAS_Week2_-PSQI_week6:_ *P_corrected_* = 0.004; VAS_Week2_-PSQI_week14:_ *P_corrected_* = 0.004; Figure 2D). Meanwhile, sleep quality during (week 1) and immediately after the treatment (week 2) was not able to predict VAS scores in the follow-up timepoints (all *P_corrected_* > 0.05).

### Safety assessment

There was no serious adverse effect by monitoring patients’ vitality, physical and mental health. There was a slight chance to experience mild headache (5, 15, 10% in the M1, DLPFC, Sham group) and/or mild scalp discomfort (15, 20, 15% in the M1, DLPFC and Sham group), but three groups showed no difference and the sensations dissolved within minutes or hours. Overall, the treatments were safe and well-tolerated by this treatment protocol (Wang et al., 2023).

## Discussion

This double-blind, randomised, sham-controlled study was designed to investigate the analgesic efficacy of motor cortex- and DLPFC-rTMS in postherpetic neuralgia. Results indicated an excellent analgesic response following motor cortex rTMS with the effect being maintained up to 3 months. This analgesic effect was supplemented by improvement in patients’ global impression of change. In addition to pain, there was also a significant improvement and remission in sleep disturbance by targeting either the motor cortex or the DLPFC, although DLPFC stimulation was not superior to sham stimulation in reducing pain sensations. We further provided interesting findings on the dynamic relationships between pain and sleep quality following motor cortex rTMS, in which pain sensations uniquely predicted improvement in sleep quality. These treatment protocols were safe with a slight chance to experience mild headache or scalp discomfort.

Our results indicated that motor cortex rTMS significantly reduced pain experience in postherpetic neuralgia, demonstrated by a consistent reduction in both VAS (Figure 2A) and SF-MPQ (Figure 2B). Meanwhile, DLPFC stimulation was not superior to sham stimulation although both groups reduced pain experience to some extent. Attal et al. (2021) have provided compelling evidence that motor cortex rTMS is able to reduce neuropathic pain but DLPFC stimulation had no clear benefits. Neuropathic pain in that multicentre, larger trial was mainly caused by traumatic/surgical nerve lesion or sensory polyneuropathy. Building on this study, our data provided further evidence that motor cortex rTMS is also superior to DLPFC stimulation in the management of postherpetic neuralgia. More importantly, the analgesic effect of rTMS was maintained up to 3 months following treatment (Figures 2A,B). This result extends previous findings in which only a short-term analgesia was observed following rTMS treatment (Che et al., 2021). Overall, our findings indicate that rTMS over the motor cortex has clear clinical relevance for postherpetic neuralgia with potential long-term analgesia.

It is also important to highlight that the response rate of pain was improved in our data compared to previous rTMS studies on postherpetic neuralgia. Previous studies reported a 40% response rate (average across different baseline pain groups) at the end of treatment (Ma et al., 2015; Pei et al., 2019). Meanwhile, our data reported an excellent 65% response rate from post-treatment extending to 3-month follow-up (Table 2). In one way, the increased response rate could result from severe pain sensations at baseline. Baseline pain was found to have a positive impact on rTMS analgesia in postherpetic neuralgia (VAS ≥7: rate = 51.95%; VAS <7: rate = 30.45%) (Ma et al., 2015; Pei et al., 2019). Our participants reported an average of 7.2 in VAS which may facilitate the analgesic effects induced by rTMS. In another way, we have doubled the dosage by delivering 3,000 (vs. 1,500) pulses per session compared to the literature on postherpetic neuralgia (Ma et al., 2015; Pei et al., 2019). Indeed, there is evidence that high-dose motor cortex rTMS is more effective than lower-dose stimulation for treating neuropathic pain (Cruccu et al., 2016; Mori et al., 2022). Unfortunately, sessions of 3,000 pulses have rarely been administrated in neuropathic pain conditions (but see Ayache et al., 2016; Che et al., 2021), although it has long been cleared by the Food and Drug Administration (FDA) for treating major depression disorders. Our findings together suggest that high-dose motor cortex rTMS may lead to excellent response particularly in postherpetic neuralgia with severe pain sensations.

In addition to pain sensations, our data also demonstrated significant benefits on sleep quality. Interestingly, both the M1 and DLPFC stimulation significantly improved sleep quality in patients with postherpetic neuralgia (Figure 2C). More importantly, both groups achieved a 60% remission rate even at 3-month follow-up (Table 2). The two previous studies on postherpetic neuralgia demonstrated a protective effect on sleep quality after motor cortex rTMS (Ma et al., 2015; Pei et al., 2019). However, sleep quality was assessed with a single question in these studies. Our study systematically evaluated sleep quality with the well-recognised Pittsburgh Sleep Quality Index (PSQI) (Buysse et al., 1989). This assessment tool also allows to analyse remission in which our data demonstrated an excellent remission rate even at 3 months following treatment. It is worth noting that Attal et al. (2021) also observed an improvement in sleep quality by both M1 and DLPFC stimulation in their large trial on neuropathic pain, although this effect was not statistically different from sham stimulation. By using a more systematic assessment tool, our findings therefore confirmed the benefits on sleep quality in patients with postherpetic neuralgia, and this effect could be achieved by either targeting the motor cortex or the DLPFC.

We also provided novel findings on the dynamic relationship between improvement in pain sensations and sleep quality following rTMS treatment. Specifically, pain sensations during and after M1 treatment were able to predict sleep quality in all the follow-up periods (Figure 2D). Meanwhile, early sleep quality was not able to predict follow-up pain sensations vice versa. It is widely accepted that patients with neuropathic pain are more likely to develop sleep disorders (Cheatle et al., 2016; Mehta et al., 2016). Our samples of postherpetic neuralgia indeed demonstrated the comorbidity of pain (VAS > 7) and sleep disturbances (PSQI > 10). Our data thus presented unique and dynamic associations between pain and sleep quality following M1 stimulation, in which improvement in sleep quality is likely to result from the improvement in pain sensations. Meanwhile, it remains unknown what led to the improvement in sleep quality in DLPFC stimulation without clear effects on pain sensations. More evidence is thus needed surrounding the mechanisms of DLPFC stimulation on sleep. Overall, these novel findings on pain-sleep dyad have direct implications for managing sleep disorders in chronic pain conditions. Moreover, these findings could have implications for improving sleep quality with rTMS beyond chronic pain as we have demonstrated the efficacy of alternative targets (Nardone et al., 2020; Lanza et al., 2023).

Patients’ Global Impression of Change (PGIC) indicated that more patients reported improvement in the M1 group compared to sham stimulation at 3-month follow-up (Figure 3A). When data were categorised based on much to very much improvement (Figure 3B), our results were consistent with the larger trial conducted in neuropathic pain (Attal et al., 2021). These findings further confirm the efficacy of motor cortex rTMS in postherpetic neuralgia as well as the importance of this self-reported, global measure of change in capturing clinically relevant pain relief (Dworkin et al., 2008). Our data on the NNT analyses revealed a 2.5 and 2.5 at post-treatment and follow-up, respectively, in the M1 group, consistent with the large neuropathic pain trial (Attal et al., 2021).

It is noted that there were no significant changes in anxiety, depression or cognition following either stimulation target (Supplementary material). These results did not deviate from the widely accepted antidepressant effects of DLPFC-rTMS in major depression disorders (Fitzgerald et al., 2003; Blumberger et al., 2018), whereby our patients were not sufficiently depressed. These results were also consistent with the larger neuropathic pain trial (Attal et al., 2021) which reported similar baseline depression scores to ours and found no effect on depression by rTMS. It is noted that there is a high comorbidity between chronic pain and depression (Von Korff et al., 1988; Bair et al., 2003). There is no conclusive evidence that TMS targeting the M1 alone in chronic pain helps with depression (Leung et al., 2020). Meanwhile, in some of the pain studies that used the DLPFC as the target for chronic pain, there was noted improvement in depressive symptoms along with chronic pain (Short et al., 2011; Leung et al., 2020; Li et al., 2022). Overall, more well-controlled studies are required with varied treatment locations and a more prolonged treatment course.

It remains an open question the mechanisms driving the analgesic effects of motor cortex rTMS (for a review see Moisset et al., 2016). It is largely accepted that motor cortex rTMS may be able to activate brain regions involved in the descending pain modulation (Lefaucheur et al., 2004; Passard et al., 2007; Onesti et al., 2013; Dall'Agnol et al., 2014). This distant and diffuse pattern of brain activation is also consistent with the presentation of diffuse and non-somatotopic analgesic effects induced by motor cortex rTMS, as demonstrated by many previous studies (Nahmias et al., 2009; Mhalla et al., 2011; Attal et al., 2021) as well as results from the current study (i.e. focal stimulation but pain relief in distinct body areas). The presence of long-term effects in our data and others (Attal et al., 2021) also indicate neuroplastic changes induced by repetitive sessions of TMS. Indeed, there are quite some studies that reported neuroplastic changes following motor cortex rTMS in neuropathic pain (Lefaucheur et al., 2006; Hosomi et al., 2013; Liu et al., 2021; Teixeira et al., 2021).

There were some limitations in this study. Patients allocated to the M1 group had a longer course of disease than patients in the DLPFC group. Nonetheless, the three groups were comparable in baseline pain, emotional assessments, as well as sleep quality. Moreover, a longer course of disease potentially indicates the refractory nature of postherpetic neuralgia, but our results demonstrated an excellent analgesic response by motor cortex rTMS anyway. Overall, the different course of disease may not challenge the results presented here but it is needed to be carefully controlled in future trials. In addition, a neuronavigation system was not feasible in our clinic. A navigation system would be able to increase targeting accuracy and consistency over the treatment courses (Ayache et al., 2016; Attal et al., 2021).

Our findings also provide insights for future studies. Accelerated forms of TMS using theta-burst stimulation (AiTBS) have recently been demonstrated to increase treatment efficacy as well as to shorten treatment durations in depression (Cole et al., 2020, 2021). These protocols represent a promising development in TMS treatment (Caulfield et al., 2022; Weissman and Daskalakis, 2022). It is expected to see AiTBS trials in neuropathic pain conditions considering the duration of rTMS protocols and medium efficacy in pain management (Lefaucheur and Nguyen, 2019). In addition to treatment protocols, this study also highlights the need for mechanistic evidence in TMS analgesia. In one way, neuroplastic evidence is limited to corticospinal pathways recorded with TMS-induced changes in electromyography, such as motor-evoked potential (MEP), short- (SICI) and long-interval intracortical inhibition (LICI; Parker et al., 2016). Concurrent TMS electroencephalography (TMS-EEG) represents a promising technology to evaluate local and distributed neuroplastic changes in the central nervous system following TMS treatment (Rogasch et al., 2014; Cash et al., 2017; Che et al., 2019). In another way, neural pathways underlying motor cortex analgesia remains to be determined and specified with functional Magnetic Resonance Imaging (fMRI). Identification of brain networks mediating rTMS analgesia would be valuable to increase treatment efficacy with functional connectivity guided, personalised targeting methodologies and treatment (Cash et al., 2020, 2021a,b).

In conclusion, in this double-blind, randomised, sham-controlled trial, motor cortex rTMS is superior to DLPFC stimulation in treating postherpetic neuralgia with excellent pain response and long-term analgesia. Meanwhile, motor cortex and DLPFC stimulation were equally effective in improving sleep quality in postherpetic neuralgia. These findings bear direct clinical implications given the refractory pain and reduced quality of life in postherpetic neuralgia.

## Data availability statement

The original contributions presented in the study are included in the article/Supplementary material, further inquiries can be directed to the corresponding authors.

## Ethics statement

The studies involving human participants were reviewed and approved by the Ethics Committee of Hangzhou First People’s Hospital (IIT-20220301-0039). The patients/participants provided their written informed consent to participate in this study.

## Author contributions

HW, YH, LY, and XC contributed to study design, data collection, data analysis and writing-up. JD, YY, and MH contributed to data analysis. All authors contributed to the article and approved the submitted version.

## Funding

XC was supported by the National Natural Science Foundation of China (4045F41120040), the Key Research and Development Programme of Zhejiang Province (2022C03038) and Hangzhou Municipal Health Commission (2021WJCY130).

## Conflict of interest

The authors declare that the research was conducted in the absence of any commercial or financial relationships that could be construed as a potential conflict of interest.

## Publisher’s note

All claims expressed in this article are solely those of the authors and do not necessarily represent those of their affiliated organizations, or those of the publisher, the editors and the reviewers. Any product that may be evaluated in this article, or claim that may be made by its manufacturer, is not guaranteed or endorsed by the publisher.

## Supplementary material

The Supplementary material for this article can be found online at: https://www.frontiersin.org/articles/10.3389/fnins.2023.1158737/full#supplementary-material
