# Supplementary figures and images for: A randomised sham-controlled study evaluating rTMS analgesic efficacy for postherpetic neuralgia
Source: Front Neurosci. 2023 May 11;17:1158737. doi: 10.3389/fnins.2023.1158737 (PMC10213647; doi:10.3389/fnins.2023.1158737)

a.

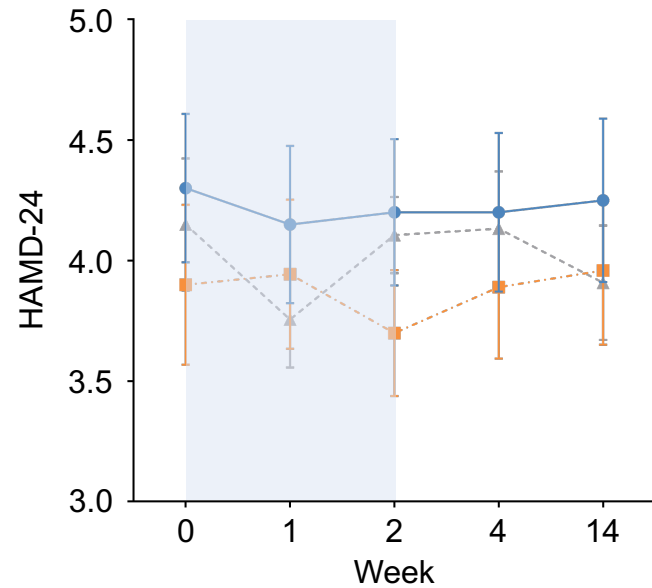

b.

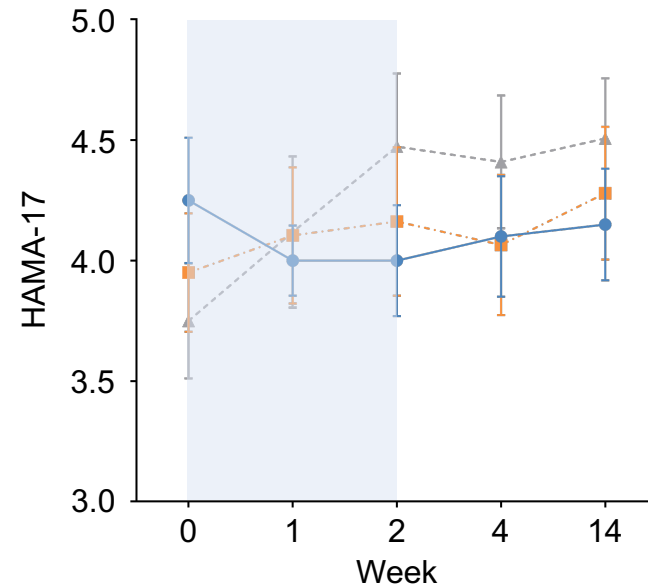

c.

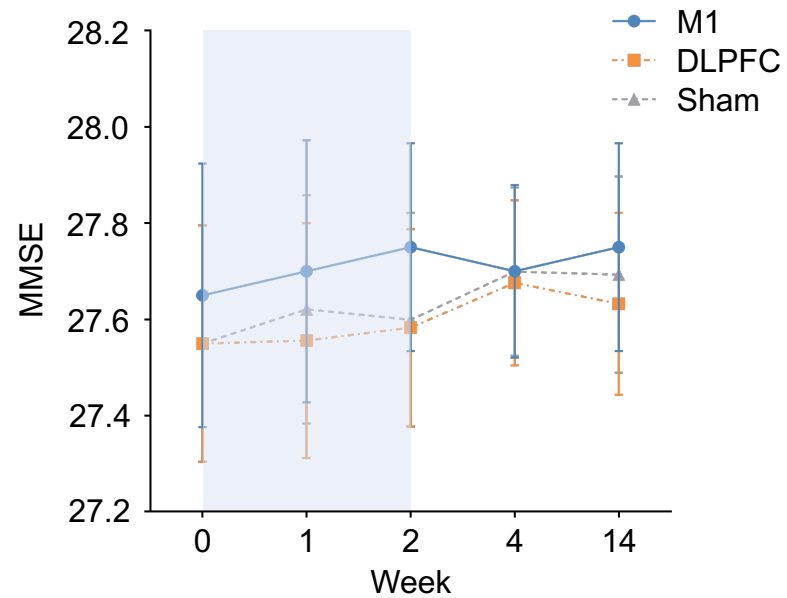

Supplement: Supplementary file 1 [file Data_Sheet_1.PDF]
